# Intermittent High Glucose Implements Stress-Induced Senescence in Human Vascular Endothelial Cells: Role of Superoxide Production by NADPH Oxidase

**DOI:** 10.1371/journal.pone.0123169

**Published:** 2015-04-16

**Authors:** Morihiko Maeda, Toshio Hayashi, Natsumi Mizuno, Yuichi Hattori, Masafumi Kuzuya

**Affiliations:** 1 Department of Geriatrics, Nagoya University Graduate School of Medicine, Nagoya 466–8550, Japan; 2 Department of Molecular and Medical Pharmacology, Graduate School of Medicine and Pharmaceutical Sciences, University of Toyama, Toyama 930–0194, Japan; University of Newcastle, UNITED KINGDOM

## Abstract

Impaired glucose tolerance characterized by postprandial hyperglycemia, which occurs frequently in elderly persons and represents an important preliminary step in diabetes mellitus, poses an independent risk factor for the development of atherosclerosis. Endothelial cellular senescence is reported to precede atherosclerosis. We reported that continuous high glucose stimulus causes endothelial senescence more markedly than hypertension or dyslipidemia stimulus. In the present study, we evaluated the effect of fluctuating glucose levels on human endothelial senescence. Constant high glucose increased senescence-associated-β-galactosidase(SA-β-gal) activity, a widely used marker for cellular senescence. Interestingly, in intermittent high glucose, this effect was more pronounced as well as increase of p21 and p16^INK4a^ , senescence related proteins with DNA damage. However, telomerase was not activated and telomere length was not shortened, thus stress-induced senescence was shown. However, constant high glucose activated telomerase and shortened telomere length, which suggested replicative senescence. Intermittent but not constant high glucose strikingly up-regulated the expression of p22*^phox^*, an NADPH oxidase component, increasing superoxide. The small interfering RNA of p22*^phox^* undermined the increase in SA-β-gal activity induced by intermittent high glucose. Conclusively, intermittent high glucose can promote vascular endothelial senescence more than constant high glucose, which is in partially dependent on superoxide overproduction.

## Introduction

Senescence of vascular endothelial cells is an important contributor to the pathogenesis of human atherosclerosis [1]. Numerous studies have shown that the senescent phenotype of endothelial cells can be transformed from anti-atherosclerotic to pro-atherosclerotic [2]. We have provided evidence that senescent endothelial cells are found in human atherosclerotic lesions but not in nonatherosclerotic lesions [3]. Diabetes mellitus is a well-documented, high-risk factor for the development of atherosclerosis [4]. The hallmark of diabetes is the presence of hyperglycemia, which can accelerate senescence in endothelial cells [5]. In several publications, we have demonstrated that high glucose can induce endothelial cell senescence [3,6,7,8]. In fact, we previously reported that continuous high glucose stimulus causes endothelial senescence more markedly than hypertension or dyslipidemia stimulus [8].

Rapid fluctuations of glycemia, including postprandial hyperglycemia and interprandial hypoglycemia, may play a crucial role in the pathogenesis of diabetic cardiovascular complications [9,10]. In clinical practice, the intensive treatment goal targets minimizing the sharp fluctuations of blood glucose levels that occur in inadequately treated patients. Intermittent, rather than constant, hyperglycemia can induce an increased production of collagen by cultured mesangial cells [11]. Furthermore, intermittent high glucose has been demonstrated to exhibit more marked increases in endothelial cell apoptosis [12,13], endothelial expression of adhesion molecules [14], and vascular smooth muscle cell proliferation [15] than constant high glucose. Thus, glucose fluctuations appear to be more deleterious to vascular cell integrity than constant high glucose concentrations.

In the present study, we verified whether intermittent vs. constant exposure to high glucose might have different effects on cellular senescence in human vascular endothelial cells. We also evaluated how fluctuating glucose makes an impact on the changes in endothelial nitric oxide synthase (eNOS) and reactive oxygen species (ROS), both of which may play pivotal roles in endothelial cell senescence [2,3].

## Materials and methods

### Cell culture

**Fig 1:**
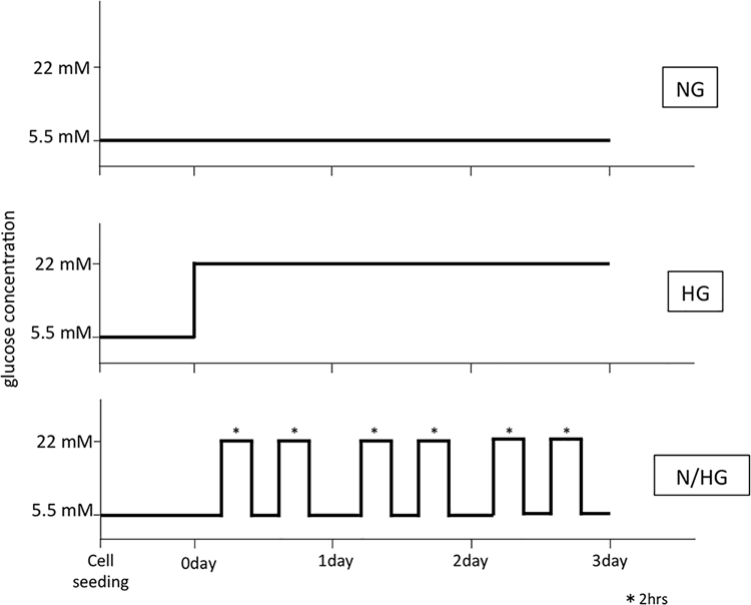
Research Design. The endothelial cells were exposed to the experimental condition for 3 days. They were grouped as follows: (1) constant normal glucose medium (5.5 mM:NG); (2) constant high glucose medium (22 mM: HG); and (3) alternating normal and high glucose media every 12 h (N/HG).

Human umbilical venous endothelial cells (HUVECs) were purchased from Lonza (Walkersville, MD, USA) and were cultured in endothelial cell growth medium-2 until the start of the experiment. To exclude the insulin effect [6], the cells were cultured in modified endothelial cell growth medium-2 that lacked insulin-like growth factor-1 but contained 2% fetal bovine serum during the experimental period. According to our previous study [3], five- to seven-passage subconfluent cells were used in the experiments. The cells were harvested at subconfluence and were seeded into six-well plates. Subsequently, they were exposed to the experimental condition for 3 days. They were grouped as follows: (1) constant normal glucose medium (5.5 mM); (2) constant high glucose medium (22 mM); and (3) alternating normal and high glucose media every 12 h (Fig 1). In the constant normal or high glucose group, each fresh medium was provided every 24 h. Mannitol was used to rule out the effect of osmotic pressure [6]. In measurements of telomere length, we continued the culture with every 3 days changing the tissue culture medium up to 28 days.

### Measurement of cellular senescence

Cytochemical staining of senescence-associated-β-galactosidase (SA-β-gal) was performed using a Senescence Detection Kit (BioVision, Milpitas, CA, USA), as previously described, which showed the detailed effect of endothelial senescence including the growth curve [6–8]. SA-β-gal activity was measured using flow cytometry [6]. Immunoblot detection was used to identify the tumor suppressor p53, which is known to be a canonical inducer of cellular senescence [16]. SA-β-gal activity was measured using a microscope [8]. The cells were fixed for 10 min in 2% formaldehyde, 0.2% glutaraldehyde in PBS and were incubated for 12 hours at 37°C without CO_2_ with fresh β-gal staining solution: 1 mg/ml 5-bromo-4-chloro-3-indolyl-b-D-galactopyranoside, 5 mM potassium ferrocyanide, 5 mM potassium ferricyanide, and 2 mM MgCl_2_, pH 6.0. The cells were counterstained with 4’6-diamidinophenylindole (DAPI; 0.2 mg/ml in 10 mM NaCl) for 10 min to count the total number of cells. The percentage of SA-β-gal-positive cells was determined by counting the number of blue cells within a sample of 1,000 cells.

Cellular senescence related proteins such as p21, p16^INK4a^ p16 and p53 were measured by western blotting. We used p21 (sc-397, SANTA CRUZ) rabbit polyclonal antibody (1:200), p16^INK4a^(ab54210, abcam) mouse monoclonal antibody (1:500), p53 mouse monoclonal antibody as well asβ-actin(ab8226, abcam) mouse monoclonal (1:15,000) as first antibody. We also used anti-rabbit IgG, HRP-Linked antibody (#7074, Cell Signaling) for p21 (1:5,000), anti-mouse IgG, HRP-Linked antibody (#7076, Cell Signaling) for p16 (1:5,000), p53(1:5,000) and β-actin (1:10,000) as 2^nd^ antibody.

We further quantificated DNA Damage of endothelial cells by using QIAamp DNA Mini Kit (51304, QIAGEN) and DNA Damage Quantification Colorimetric Kit (#K253-25, Bio Vision). Apurinic/apyrimidinic(AP) sites are one of the major types of DNA lesions formed during the course of base excision and repair of oxidized, deaminated or alkylated bases. It has been estimated that about 2×10^5^ base lesions are generated per cell per day. The level of AP sites in cells can be good indicator of DNA lesion and repair against chemical damage and cellular senescence. This Kit utilizes the ARP (Aldehyde Reactive Probe) reagent that reacts specifically with an aldehyde group, which is the open ring from of the AP sites. After treating DNA containg AP sites with ARP reagents, AP sites are tagged with biotin resideues, which can be quantified using avidin-biotin assay followed by a colorimetric detection.

### Human telomerase activity and length

Telomerase activity was measured using the TeloTAGGG Telomerase PCR ELISA^PLUS^ kit (Roche Diagnostics, Mannheim, Germany) [6]. Telomere length was measured via fluorescence in situ hybridization using flow cytometry [6, 17]. For the assay standardization, we added an aliquot of a single batch of 1,301 cells (a sub-line of human T cell lymphoblast-like cell) to each sample as an internal control.

### Western blot analysis

Western blotting was performed as described in our previous reports [7,8,18]. The membrane was blotted with the indicated antibodies and was processed using chemiluminescence. Namely, we used p21 (sc-397, SANT CRUZ) rabbit polyclonal (1:200), p16^INK4a^(ab54210, abcam) mouse monoclonal (1:500) and β-actin(ab8226, abcam) mouse monoclonal antibodies (1:15,000) as 1^st^ antibodies and anti-rabbit IgG, HRP-Linked antibody (#7074, Cell Signaling) for p21 (1:5,000) anti-mouse IgG, HRP-Linked antibody (#7076, Cell Signaling) for p16 (1:5,000) and β-actin (1:10,000) antibodies as 2^nd^ antibodies.

### DNA Damage Quantification

To detect DNA damage quantification, we used QIAamp DNA Mini Kit (51304, QIAGEN) And DNA Damage Quantification Colorimetric Kit (#K253-25, Bio Vision). Apurinic/apyrimidinic(AP) sites are one of the major types of DNA lesions formed during the course of base excision and repair of oxidized, deaminated or alkylated bases. It has been estimated that about 2×10^5^ base lesions are generated per cell per day. The level of AP sites in cells can be good indicator of DNA lesion and epair against chemical damage and cell aging. This Kit utilizes the ARP (Aldehyde Reactive Probe) reagent that reacts specifically with an aldehyde group, which is the open ring from of the AP sites. After treating DNA containing AP sites with ARP reagents, AP sites are tagged with biotin resideues, which can be quantified using avidin-biotin assay followed by a colorimetric detection.

### Nitric oxide and metabolites of nitric oxide measurement

To detect nitric oxide (NO) production by HUVECs, we loaded HUVECs with 4,5-diaminofluorescein diacetate (DAF-2 DA, Cayman Chemical, San Diego, CA, USA). This membrane-permeable dye is hydrolyzed intracellularly by cytosolic esterases releasing DAF-2, which is converted in the presence of NO into a fluorescent product, DAF-2 triazole [19].

The metabolites of NO (NOx:NO2^-^ and NO3^-^) in the medium were measured using the HPLC system to develop NOx measurement [20].

### Analysis of ROS and superoxide generation

To detect intracellular ROS, including hydrogen peroxide, hydroxyl radical, and peroxynitrite, we loaded the endothelial cells using the fluorescent probe 5-(and-6)- chloromethyl-2’,7’-dichlorodihydrofluorescein diacetate, acetyl ester (CM-H_2_DCFDA) (Invitrogen, Carlsbad, CA, USA) to produce a final concentration of 10 mM for 45 min in phosphate buffered saline at 37°C. At the end of the experiment, the cells were rinsed with phosphate-buffered saline and were subsequently placed in the culture medium. Imaging was conducted using a Cell Observer microscope and AxoVision software (Carl Zeiss, Oberkochen, Germany) [18]. The production of superoxide anion was speculated from measurements using dihydroethidium (DHE) (Invitrogen) [21–23]. The cells were incubated using fluorescent probes at the concentration of 10 mM at 37°C for 30 min, and flow cytometry was performed [8].

### Transfection of siRNAs

All small interfering RNAs (siRNAs) were purchased from Santa Cruz Biotechnology (Santa Cruz, CA, USA). The nonsilencing control siRNA was used as a negative control. Scrambled siRNA-treated cells were also used as controls. The transfection of siRNA was performed in Opti-MEM I Reduced-Serum Medium using Lipofectamine RNAiMAX (Invitrogen) [24]. We confirmed the expression of a target gene by Western-blot. There are differences in the suppression ratio (namely reflecting the transfection efficiency) according to target genes. In siRNA of eNOS, the suppression ratio was approximately 99%. In siRNA of p22phox, it was 96.5%. There is a report that the transfection reagent (RNAiMAX), which we used in the present study, shows the high transfection efficiency in smooth muscle cell as adherent cell, too [25].

### Statistics

The values are presented as the mean ± standard deviation (SD). One-way ANOVA followed by Tukey’s multiple comparison test was used, and *p*<0.05 was considered to be statistically significant.

## Results

**Fig 2:**
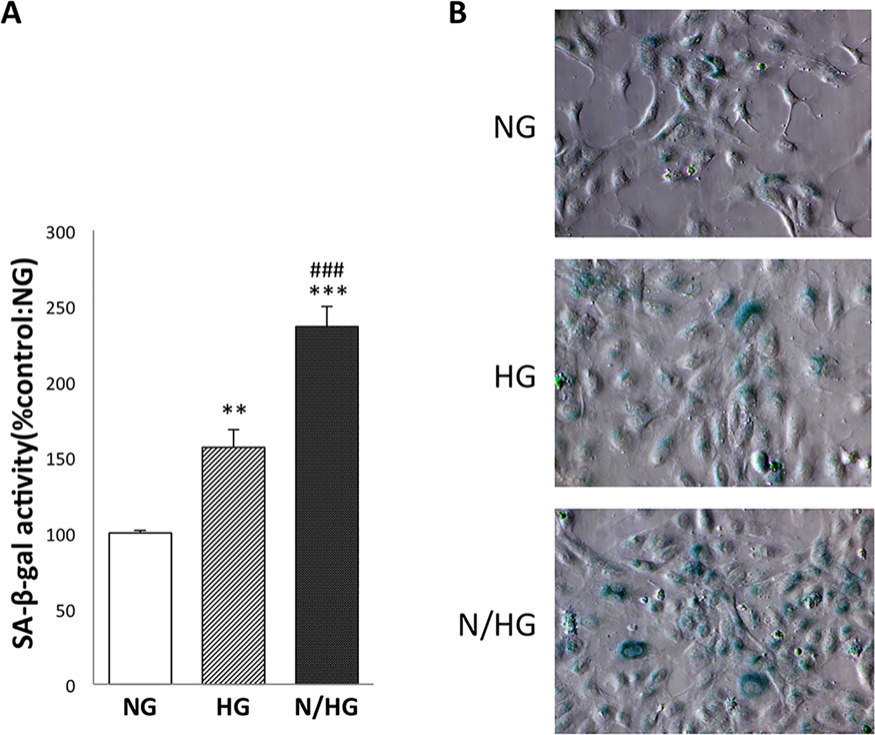
Effect of high glucose on SA-β-gal activity in HUVECs. NG, constant normal glucose (5 mM); HG, constant high glucose (22 mM); and N/HG, 5 mM alternating with 22 mM glucose. To confirm the effect of glucose in HUVECs, NG, HG and N/HG were cultured with each Stimulus for 3 days. HUVECs were cultured with constant high glucose (HG) and intermittent glucose (N/HG) introduced in Fig 1. Namely, N/HG was stimulated twice with HG (at 4-hour intervals) for a total of 4 hours daily (9 a.m. to 11 a.m., 3 p.m. to 5 p.m.), and was cultured in NG in other time of the total 4-hour HG stimulation. (A) SA-β-gal activity was evaluated cytochemically. The values of the three independent experiments are mean ± S.D. **p<0.01; ***p<0.001 vs. NG; ###p<0.001 vs. HG. (B) SA- β-gal-positive cells (blue) can be detected via cytochemical staining.

When HUVECs were exposed to constant high glucose, there was a significant increase in SA-β-gal activity, a widely used quantitative marker for aging *in vitro*. This effect was enhanced when the cells were exposed to intermittent high glucose concentrations (Fig 2A). Similarly, cytochemical staining SA-β-gal showed that the number of SA-β-gal positive cells showed that the number of SA-β-gal positive cells was induced by the high-glucose condition and was increased under the fluctuating-glucose condition (Fig 2B). SA-β-gal activity was not affected by osmolality, which was confirmed by the lack of effect of mannitol, as reported in our previous study [6].

**Fig 3:**
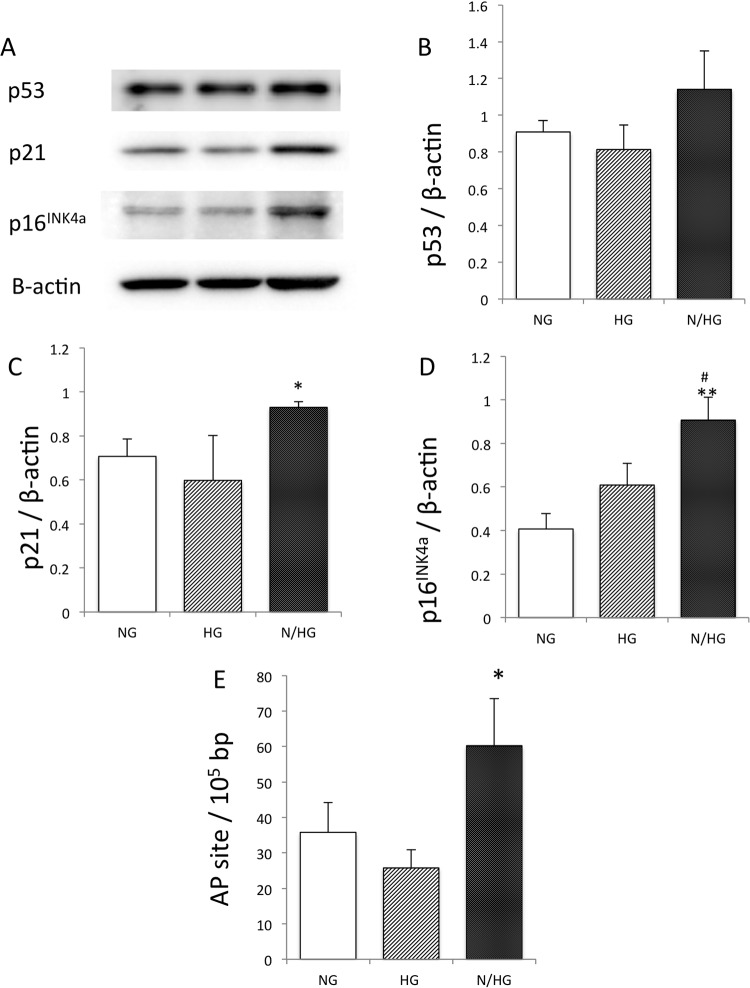
Effect of high glucose on p53, p21, p16^INKa^, and DNA ladder on Apurinic/apyrimidinic (AP) sites. HUVECs were cultured with constant high glucose (HG) and intermittent glucose (N/HG) introduced in Fig 1. Namely, N/HG was stimulated twice with HG (at 4-hour intervals) for a total of 4 hours daily (9 a.m. to 11 a.m., 3 p.m. to 5 p.m.), and was cultured in NG in other time of the total 4-hour HG stimulation. (A)-(D): Effect of high glucose on p53, p21, p16^INKa^ protein. (E): Effect of continuous and intermittent high glucose on endothelial DNA damage on Apurinic/apyrimidinic(AP) sites. *p<0.05; **p<0.01 vs. NG; #p<0.05 vs. HG. The values of the three independent experiments are mean ± S.D.

Endothelial senescence related proteins such as p53, p21, p16^INK4a^ and were measured by western blotting (Fig 3A, 3B, 3C and 3D). Intermittent high glucose stimulus (IHG) increased these proteins significantly compared with the condition of normal glucose and tended to increase them than those by continuous high glucose stimuli. We further quantificated DNA damage of endothelial cells. DNA damage was observed by the stimuli of inthermittent high glucose (Fig 3E) as well as continuous high glucose stimuli. These results show that intermittent high glucose condition induced endothelial senescence, which starts as early as 3 days after the start of the stimuli.

**Fig 4:**
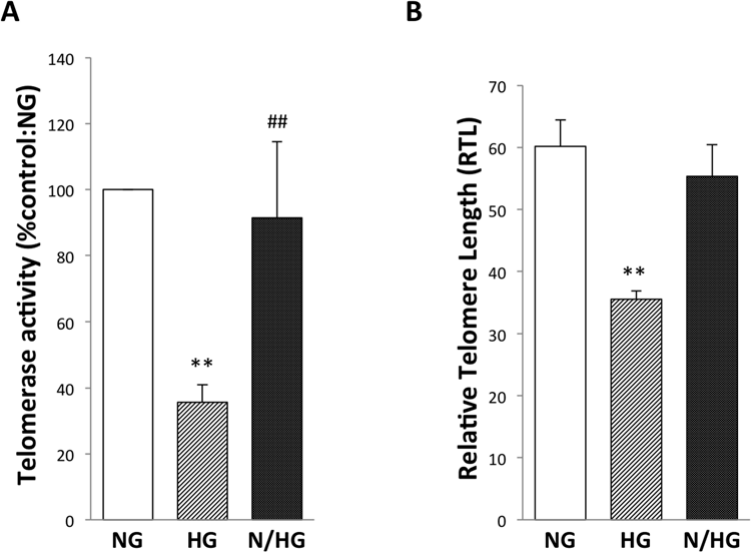
Effect of high glucose on telomerase activity and telomere length in HUVECs. NG, constant normal glucose (5 mM); HG, constant high glucose (22 mM); and N/HG, 5 mM alternating with 22 mM glucose. (A) Telomerase activity was measured by the telomere repeat application protocol (trap) assay. The values of the three independent experiments are mean ± S.D. **p<0.01 vs. NG; ##*p*<0.01 vs. HG. (B) Telomere length was measured to evaluate the relationship to replicative senescence. The data shown represent the average of two independent experiments.

The telomerase, a specialized ribonucleprotein transcriptase that is important for long-term eukaryotic cell proliferation and genomic stability to replenish the DNA at telomeres, was significantly decreased in a constant high-glucose environment (Fig 4A). Subsequently, telomere length, a likely index of biological aging, was shortened by 4 weeks, which we continued the culture with every 3 days changing the tissue culture medium up to 28 days. It suggested replicative senescence (Fig 4B). In contrast, both telomerase activity and telomere length remained unchanged after exposure of HUVECs to intermittent high glucose (Fig 4A and 4B).

**Fig 5:**
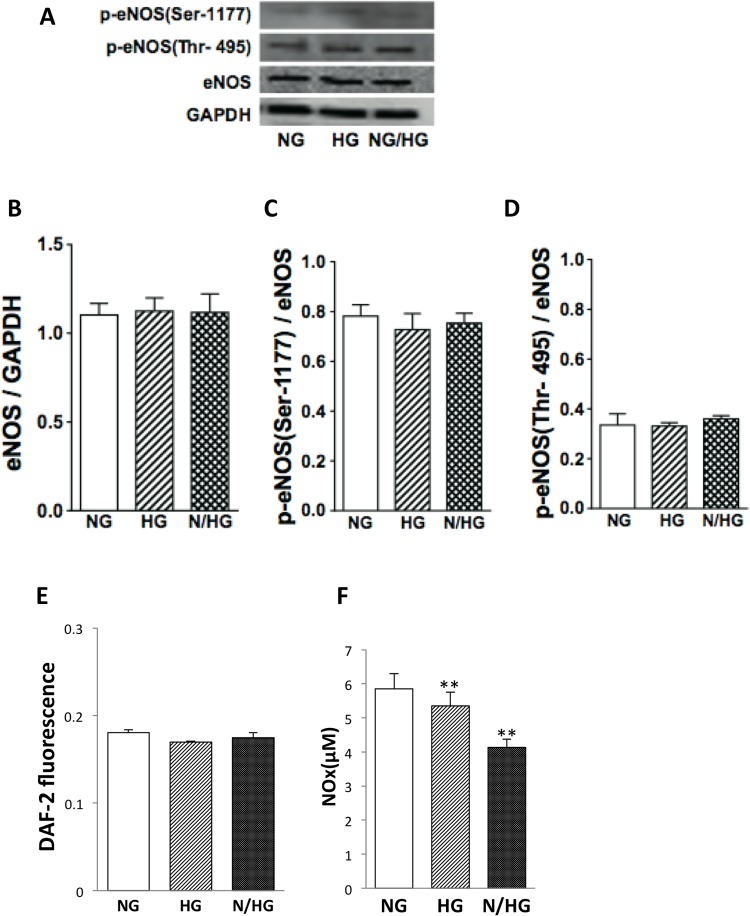
eNOS activity in HUVECs exposed to high glucose. NG, constant normal glucose (5 mM); HG, constant high glucose (22 mM); and N/HG, 5 mM alternating with 22 mM glucose. (A) Typical Western blots for total eNOS, Ser-1177 phosphorylated eNOS, and Thr-495 phosphorylated eNOS are shown. GAPDH served as the loading control. (B-D) Summary of quantification of densitometric measurement of the immunoblot data. Total eNOS expression and eNOS phosphorylation levels were normalized to GAPDH and total eNOS, respectively. (E) NO production by HUVECs, using the fluorescent dye DAF-2-DA. (F) The data of NOx (NO2^-^ and NO3 ^-^), NO metabolites measured by HPLC.

We have previously shown that eNOS plays a pivotal role in the regulation of the senescence program in vascular endothelial cells [2,6,7,8]. Under constant or intermittent high glucose, however, eNOS total expression, Ser-1177 eNOS phosphorylation, and Thr-495 eNOS dephosphorylation levels were substantially similar to those under normal glucose (Fig 5A–5D). When we examined the basal NO levels from HUVECs using the fluorescent dye DAF-2 DA, no significant difference was found among the three groups (Fig 5E). Concentration of NOx; metabolites of NO, NO2^-^ + NO3^-^, in culture medium also supports those data (Fig 5F).

**Fig 6:**
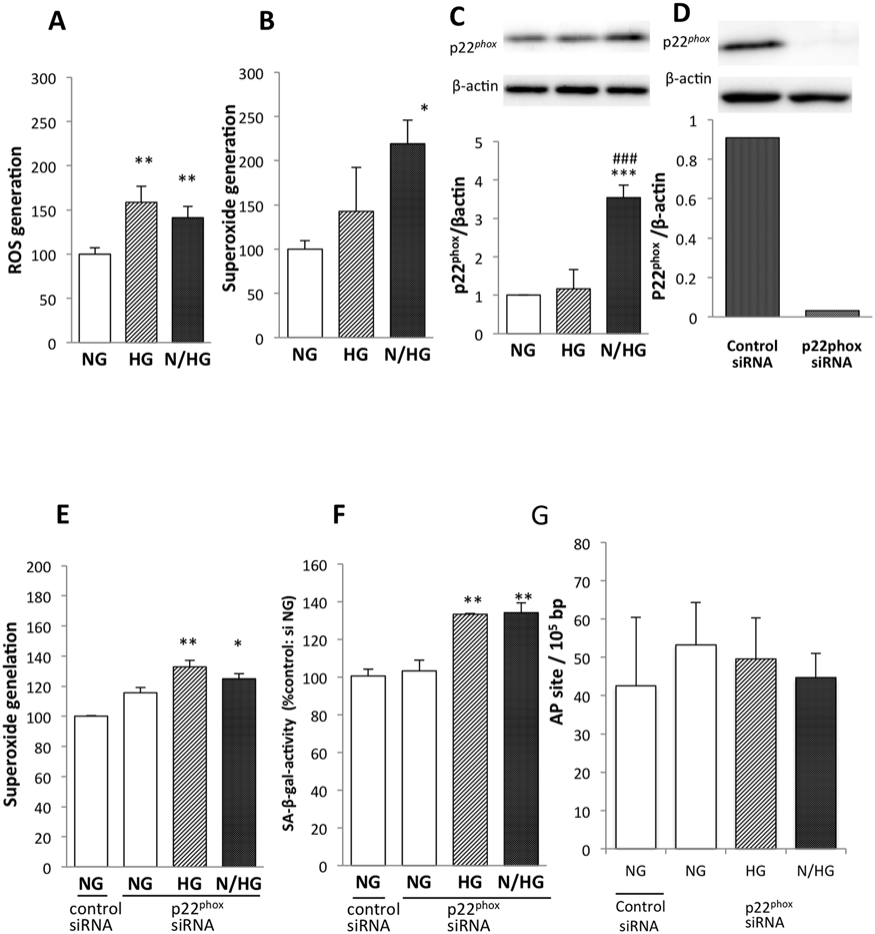
ROS and superoxide generation in HUVECs exposed to high glucose. NG, constant normal glucose (5 mM); HG, constant high glucose (22 mM); and N/HG, 5 mM alternating with 22 mM glucose. (A) Intracellular ROS was measured by visualizing the use of the fluorescent probe CM-H_2_DCFDA. (B) Superoxide was detected via DHE and was analyzed using flow cytometry. (C) Expression of p22^*phox*^ protein levels. In the top, typical Western blots are shown. β-Actin served as loading control. (D) Transfection of p22^*phox*^ siRNA effectively eliminated p22^*phox*^ protein expression. (E) Transfection of p22^*phox*^ siRNA negated the increase in superoxide production in the fluctuating-glucose condition. (F) Transfection of p22^*phox*^ siRNA blunted the fluctuating glucose-induced SA-β-gal activity. (G) Transfection of p22^*phox*^ siRNA blunted DNA damage of APsite. The values of the three independent experiments are mean ± S.D. *p<0.05; **p<0.01; ***p<0.001 vs. NG; ###*p*<0.001 vs. HG.

Our previous studies have demonstrated that increased ROS plays a critical role in endothelial cell senescence caused by high-glucose stimuli [2,6,7,8]. ROS production was significantly increased by both constant and intermittent high glucose concentrations. The extent of increased ROS production was not different between constant and intermittent high glucose (Fig 6A). When superoxide was measured using the oxidative fluorescent dye DHE, a higher increase in superoxide production was observed under intermittent high-glucose conditions compared with constant high-glucose conditions (Fig 6B). NADPH oxidase is one of the most important sources of superoxide in vascular cells and p22^*phox*^ is a critical component of the superoxide-generating NADH/NADPH oxidase system [20, 25, 26]. Constant exposure to high glucose did not alter the expression level of p22^*phox*^; however, intermittent exposure to high glucose resulted in a striking up-regulation of p22^*phox*^ expression (Fig 6C). To explore the involvement of p22^*phox*^ up-regulation in the senescent action of glucose fluctuations in endothelial cells, we used siRNA to specifically ablate p22^*phox*^ mRNA in HUVECs. Our transfection of p22^*phox*^ siRNA effectively silenced the endothelial expression of p22^*phox*^ compared with that of the negative control siRNA (Fig 6D). Under the transfection of p22^*phox*^ siRNA, there was no difference in superoxide production between cells that were exposed to normal, constant high, or intermittent high glucose (Fig 6E). Furthermore, the increase of SA-β-gal activity observed in cells exposed to oscillating glucose was significant but less pronounced when p22^*phox*^ siRNA was transfected (Fig 6F, compared with Fig 2A). Under the transfection of p22^*phox*^ siRNA, there was no difference in DNA damage in AP site between cells that were exposed to normal, constant high, or intermittent high glucose (Fig 6G).

## Discussion

Endothelial cell dysfunction is identified to be an early culprit in causing the development of vascular complications in diabetes [26, 27]. Endothelial cell senescence causes endothelial dysfunction [1]. Thus, endothelial cell senescence may be involved in diabetes-related vascular disorders. Our recent studies have provided increasing evidence that high glucose accelerates endothelial cell senescence [3,6,7,8].

The present study showed that human vascular endothelial cells exposed to high glucose concentrations increased cell senescence, which was determined by measuring SA-β-gal activity. This evidence was more enhanced in cells that were exposed to intermediate rather than constant high glucose concentrations. Further, p16^INK4a^ and p21were increased by 3 days intermittent high glucose stimuli. Although increase of p53 was not large compared with those of p16^INK4a^ and p21, the pathway via p16INK4a may be main process of cellular senescence, on the other hands, p53 relates apoptosis more than senescence [28]. Our findings may suggest that glucose fluctuation appears to be more deleterious to endothelial cells than a constant high glucose concentration and it may accelerate endothelial senescence within even 3 days.

As reported earlier [6,8], telomerase activity was markedly decreased when HUVECs were exposed to a constant high glucose concentration, and thus, presumably resulted in shortened telomeres. However, telomere extension by the overexpression of telomerase does not affect stress-induced senescence [29] but prevents replicative senescence [30]. Therefore, the change in telomerase activity, subsequent to the change in telomere length induced by a constant high glucose concentration may represent replicative senescence. We studied telomere activity by 3 days observation, however, in measurements of telomere length, we continued the culture with every 3days changing the incubation fluid up to 28 days to observe significant change of telomere length. It needs much longer time to observe change of the telomere length compared with the change of telomerase activity. Intermittent high glucose did not alter telomerase activity and telomere length. Telomerase activity, subsequently telomere length might be affected by ROS as below. It is reported that shorter telomeres with high telomerase activity in unstimulated leukocytes were associated with reduced social support and greater early life adversity [30]. The effect of telomerase might be complicated.

We have shown a pivotal role of increased ROS in endothelial cell senescence caused by high glucose stimuli [3,6,7,8]. In this study, no difference in ROS production was found between the cells exposed to constant and intermittent high glucose. The detection of ROS was performed after staining HUVECs with CM-H_2_DCFDA, which is now used for monitoring intracellular hydrogen peroxide, hydroxyl radical, and peroxynitrite production in cells. Thus, the enhanced cell senescent effect of glucose fluctuation cannot be attributed to the increased production of these radicals. When DHE, which is generally speculated to be specific for superoxide, was used as its probe, we found a larger production of superoxide in the cells incubated with oscillating glucose than in the cells with constant high glucose. There may be the limitation of the DHE method for the correct measurement of superoxide, however there are no perfect methods for that easily available except an ESR (electron spin resonance) method [21–23]. The endothelial expression of p22^phox^, a critical component of the superoxide-generating NADH/NADPH oxidase system, was strikingly up-regulated under intermittent high-glucose conditions. Importantly, the ablation of p22^phox^ by siRNA abrogated the increase in superoxide production and undermined the implementation of endothelial cell senescence in a fluctuating-glucose environment. We thus suggest that glucose fluctuations display a more adverse effect on senescence in human vascular endothelial cells than constant high glucose concentrations, in part, by NADPH oxidase-derived superoxide overproduction. Nox2, referred to as gp91phox, likely plays a role, and the hetero-dimer with p22phox may be produced and may increase catalytic activity. Nox4 may play a role and may limit the actual catalytic activity [31, 32]. However, in this experiment, we did not evaluate the identification of the Nox2/ Nox4 ratio because our primary object was to investigate the effect of intermittent high glucose (models of impaired glucose tolerance and metabolic syndrome) on endothelial senescence. Similarly, mitochondria may produce ROS as well as NADPH oxidase, however, we could not investigate the source of ROS in detail in the present study.

Our recent study has demonstrated that the antisenescence effect of calcium-channel blockers in human endothelial cells is associated with increased eNOS activity [8], implying that eNOS activation is important in the regulation of the senescence program in endothelial cells by NO-mediated delay of cellular senescence [2]. The activity of eNOS is regulated by reciprocal phosphorylation of the activating site Ser-1177 and inhibiting site Thr-495, which affects NO bioavailability [33]. We found that both constant and intermittent glucose led to no changes in eNOS total expression, Ser-1177 eNOS phosphorylation, Thr-495 eNOS dephosphorylation, or NO release from control levels in HUVECs. This finding suggests that the baneful effect of glucose fluctuation on endothelial cell senescence cannot be attributed to a substantial alteration in eNOS activation. Several papers have reported the expression of iNOS by high glucose in endothelial cells; however, the glucose concentration in those papers was higher (33 mM or more) than those in our finding (22 mM)[34,35], and inducers of iNOS, such as lipopolysaccharides, were simultaneously used in several experiments. Other papers have reported no induction of iNOS by high glucose in the cultured endothelium [36]. In fact, we have observed iNOS in neighboring necrotic areas in atherosclerosis but not in the endothelium in diabetic patients [37]. Altogether, we believe that iNOS is not induced by high glucose. The NOx amount was not increased in the present study and we did not detect iNOS under similar conditions [3,7]. We speculate that one reason is the non-use of IGF in the culture medium to rule out the effect of insulin in our study [6].

The present results may be clinically relevant. Our findings support a prior clinical report that fluctuations in blood glucose levels cause endothelial dysfunction in diabetic patients [38]. Additionally, our findings might support the hypothesis that impaired glucose tolerance causes atherosclerosis via stress-induced senescence. Thus, glucose fluctuation in blood can be a risk predictor for the occurrence and the progression of cardiovascular diseases in diabetes. Appreciably, turning up highly promising management of postprandial hyperglycemic spikes is an important determinant of overall glucose control to prevent the onset and the development of hyperglycemic or diabetic cardiovascular complications.

## Conclusion

Impaired glucose tolerance occurs frequently in the elderly and poses an independent risk for atherosclerosis. Endothelial cellular senescence precedes atherosclerosis. Constant high glucose levels increased SA-β-gal activity, a cellular senescence marker, and showed replicative senescence. This effect was more pronounced in intermittent high glucose, which demonstrated a stress-induced senescence. Intermittent, but not constant, high glucose strikingly up-regulated the expression of p22^*phox*^, an NADPH oxidase component, increasing superoxide. Intermittent high glucose promotes vascular endothelial senescence and, in part, depends on superoxide overproduction.
